# Testing fungus impregnated cloths for the control of adult *Aedes aegypti* under natural conditions

**DOI:** 10.1186/1756-3305-6-256

**Published:** 2013-09-08

**Authors:** Adriano R Paula, Aline T Carolino, Carlos P Silva, César R Pereira, Richard I Samuels

**Affiliations:** 1 Department of Entomology and Plant Pathology, Universidade Estadual do Norte Fluminense Darcy Ribeiro, Campos dos Goytacazes RJ CEP 28013-602, Brazil; 2 Departamento de Bioquímica, Universidade Federal de Santa Catarina, 88040–900, Florianópolis, Brazil

**Keywords:** Virulence, Field testing, Fungus, Insecticide, Vector, Dengue, Blood feeding, Behaviour

## Abstract

**Background:**

Entomopathogenic fungi could be useful tools for reducing populations of the dengue mosquito *Aedes aegypti*. Here the efficiency of fungus (*Metarhizium anisopliae*) impregnated cloths (with and without imidacloprid [IMI]) was evaluated against adult *A. aegypti* in simulated human dwellings. Behaviour of mosquitoes in the presence of black cloths was also investigated.

**Findings:**

When mosquitoes were released into the test rooms, the lowest survival rates (38%) were seen when five black cloths impregnated with conidia of ESALQ 818 + 10 ppm IMI were fixed under tables and chairs. This result was significantly lower than the survival rate recorded when cloths were impregnated with ESALQ 818 alone (44%) or ESALQ 818 + 0.1 ppm IMI (43%). Blood fed *A. aegypti* had lower landing frequencies on black cloths than sucrose fed insects during the first 24 h following feeding, which may have been due to reduced flight activity. Few mosquitoes (4-5%) were observed to land on the cloths during the hours of darkness. The landing pattern of sucrose-fed mosquitoes on non-treated and fungus-treated cloths was similar.

**Conclusion:**

The synergism between *M. anisopliae* and IMI significantly reduced *Aedes* survival in simulated field conditions. The use of fungus impregnated cloths is a promising point source application method for the control of adult *A. aegypti*.

## Findings

The mosquito *Aedes aegypti* is the principal vector of dengue fever and conventional control methods are failing to prevent epidemics throughout the world [1]. *A. aegypti* adults are controlled during dengue epidemics using a variety of outdoor chemical spraying methods, which has now lead to the development of insecticide resistance to carbamates, organochlorines, organophosphates and pyrethroids in many regions of Brazil [2]. Therefore alternatives are urgently needed. One such alternative is the use of entomopathogenic fungi for biological control of adult *A. aegypti*. The virulence of entomopathogenic fungi against adult *A. aegypti* was recently confirmed [3,4]. Since then other studies have been carried out to investigate the virulence of entomopathogenic fungi against different life stages of *A. aegypti*[5,6]. With the aim of further reducing the survival rates of adults exposed to entomopathogens, the combination of fungi with low concentrations of conventional insecticides has been investigated. The neonicotinoid insecticide imidacloprid (IMI) has been shown to act synergistically with the fungus *Metarhizium anisopliae*, significantly reducing survival rates of sucrose-fed female *A. aegypti* exposed to both agents when compared to insects exposed to the agents separately [7]. Synergism was also confirmed for blood-fed Rockefeller and wild strain insects that had been exposed to both agents [8]. This result was important as studies have previously shown that blood-fed *Anopheles gambiae* were less susceptible to fungal infection than their sucrose fed counterparts during a 72 h post-feeding period [9] and for up to 96 h post-feeding in the case of *A. aegypti*[10].

An effective delivery system for these fungi is of utmost importance. The use of fungus impregnated black cotton cloths has been shown to be effective at reducing adult *Aedes* survival under field conditions when tested in large cages [4,8]. Other delivery systems, such as impregnating clay water storage pots with fungal spores [11] that mosquitoes are attracted to as resting sites, or applying fungi to house screens, such as eave curtains, could also be promising delivery tools for infecting mosquitoes that come into contact with these screens during host seeking behaviour [12].

In the current study we tested the effectiveness of fungus impregnated black cloths in simulated human residences as the next step in the process of developing an integrated vector management program for the control of adult *A. aegypti*. Experiments were also carried out to observe the behaviour of mosquitoes in the presence of black cloths with and without fungus.

## Methods

### Maintenance of insect colonies

*Aedes aegypti* (Rockefeller strain) colonies were reared in cages at 25°C; 75% RH; 16:8 L/D photoperiod and provided with a 10% sucrose solution. Insects were provided with blood meals by placing a mouse, immobilized in a wire mesh bag, in the adult mosquito cages (approved by the UENF Ethical Committee). Following the blood meal, oviposition occurred in beakers half filled with water and lined with filter paper placed in adult cages. Larval eclosion was stimulated by total immersion of the filter paper in water to which mouse food had been previously added (24 h) to reduce oxygen levels.

Larvae were maintained in plastic trays (80 larvae per 100 mL) and fed on freshly ground commercial mouse food (0.05 g per L) until reaching the pupal stage. Pupae were separated into water filled beakers and transferred to cages before adult emergence. Recently emerged (2–3 days old) females that had been maintained in cages with males, were separated for use in all experiments.

### Fungal isolate and preparation of suspensions

The isolate of *M. anisopliae* used here was obtained from the collection at the Escola Superior de Agricultura “Luiz de Queiroz” (ESALQ 818) in Piracicaba (São Paulo), which had been previously demonstrated to have high virulence against adult *A. aegypti*[4]. Fungi were cultured on Sabouraud Dextrose Agar (Dextrose 10 g; Peptone 2.5 g; Yeast Extract 2.5 g; Agar 20 g in 1 L H_2_0) at 27°C for 15 days before being used in experiments. Conidia were harvested directly from the solid media using a spatula and suspended in Tween 80 (0.05% in sterile distilled water). Conidial concentration was determined using a Neubauer hemocytometer. A final concentration of 1×10^9^ conidia mL^-1^ was prepared by serial dilution. Viability tests were carried out by plate counting and only batches with >90% germination were used in experiments.

### Simulated intra-domicile experiments using black cloths impregnated with fungus or fungus + IMI

Two identical rooms in the insectary of the State University of North Fluminense (each room: 4 (L) × 3 (W) × 2.8 (H) m) with ceramic floor tiles, painted walls and a metal framed window (120 × 40 cm) were used for these experiments (Additional file 1). Furniture was placed in each room: 2 chairs, 2 desks and a metal book stand. The rooms were prepared for experiments by cleaning with disinfectant and three wick feeders placed in each room with 30 mL 10% sucrose. Each wick feeder was placed in the middle of water filled Petri dish to avoid attracting domestic ants. For each experiment, previously autoclaved black cotton cloths (20 × 10 cm) were immersed in suspensions of ESALQ 818 alone (1×10^9^ conidia mL^-1^), ESALQ 818 + IMI 0.1 ppm or ESALQ 818 + 10 ppm IMI. Control treatment cloths were immersed in either Tween 80, Tween 80 + 0.1 ppm IMI or Tween 80 + 10 ppm IMI. The cloths were then left to dry for 16 h in a controlled temperature room (26°C, 71 ± 7% RH and 12 h L:D cycle). The final concentration of conidia on the cloths was approximately 1×10^8^ conidia cm^2^, estimated by removing 3 × 1 cm^2^ squares of the cloths, re-suspending the conidia in Tween by vortex mixing and then the conidial concentration was quantified using a Neubauer hemocytometer.

Pilot experiments were carried out using 3 cloths per room, which resulted in no significant differences in survival when comparing control and fungus treatments (data not shown). Therefore all further experiments were performed using 5 cloths per room.

For each experiment 5 cloths were fixed under furniture in each room using “silver tape” (3 M Ltd, Brazil). One room was used for fungus impregnated cloths whilst the other room was used for “control” treatments (Tween only, 0.1 or 10 ppm IMI). Fifty female *A. aegypti* were then released into each room and the doors sealed with masking tape. On the seventh day of the experiment, a trap (BG-Sentinel™ Biogents Ltd. Germany) was quickly placed in each room and 48 h later the number of captured mosquitoes was recorded. The rooms were then inspected for any mosquitoes that had failed to be captured. Each experiment was carried out three times, thus the survival of a total of 150 mosquitoes was monitored for each treatment. Data-loggers (Watch-Dog, USA) were used to monitor temperature and humidity hourly during experiments (temperature max: 28.8°C min: 25.7°C; RH max: 78.4% min: 61.7%). A one-way analysis of variance (ANOVA) was used to compare the survival of mosquitoes following the different treatments. When a significant effect of group was recorded, data were further analysed by Duncan’s post-hoc test with p < 0.05 as the criterion for statistical significance.

The use of 7 fungus impregnated cloths per room was also tested; however, there was no significant reduction in survival when compared to the use of 5 cloths (data not shown).

### Behaviour of mosquitoes fed on different diets in the presence of black cloths

A black cotton cloth (20 × 10 cm; previously autoclaved) was fixed to the side of a Perspex observation chamber (100 × 51 × 61 cm; see Additional file 1) using transparent tape. The chamber was placed in a room with a window (120 × 40 cm) under natural light and temperature conditions, monitored using a data-logger (temperature max: 26.9°C min: 23.8°C; RH max: 85% min: 71.6%). The rooms received direct sun-light from approximately 12:00 h to 16:00 h. For each experiment, fifty blood-fed female *A. aegypti* or fifty sucrose fed females were released into the chamber shortly after they had been offered a nutrient source. Only blood-engorged females were used for the blood fed group. At each time period (0, 2, 4, 8, 16, 24 and 32 h), an observer carefully and quickly entered the room and counted the number of mosquitoes that were resting on the cloth. During the night time observations, the lights were briefly switched on to enable more accurate counting. For each feeding regime, the experiment was performed three times. A two-way analysis of variance (ANOVA) was used to compare the number of mosquitoes resting on black cloths following the different treatments: determining the group effect, time effect, as well as the interactions between variables. When a significant effect of group versus day interaction was recorded, data were further analysed by independent *t*-test and one-way ANOVA followed by Duncan’s post-hoc test with p < 0.05 as the criterion for statistical significance.

### Behaviour of mosquitoes in the presence of black cloths impregnated with fungus

The same experiment as above was carried out except that the black cloths were previous impregnated with conidial suspensions prepared as described for the simulated intra-domicile experiments. Black cotton cloths (20 × 10 cm) were previously autoclaved and then immersed in conidial suspensions (1×10^9^ conidia mL^-1^). The cloths were then left to dry for 16 h in a controlled temperature room (26°C, 71 ± 7% RH and 12 h L:D cycle). The final concentration of conidia on the cloths was approximately 1x10^8^ conidia cm^2^. Fifty sucrose-fed insects were released into the observation chambers. Observations were performed as described above. This experiment was carried out three times.

## Results and discussion

New approaches are urgently needed to reduce the incidence of insect vectored diseases. Although the use of entomopathogenic fungi as novel biological control agents of adult mosquitoes appears promising under laboratory and simulated field conditions, extensive research is needed to effectively deliver these agents.

Simple approaches to delivering fungal biological control agents to adult mosquitoes could be highly effective and cost efficient. Here we show under field conditions that black cotton cloths impregnated with fungal conidia, when deployed in the presence of mosquitoes, significantly reduced the survival of female *A. aegypti*. Black surfaces are known to attract a range of mosquito species including *A. aegypti*[13]. Our previous results using fungus impregnated black cotton cloths in large cages [4,8] confirmed that female *A. aegypti* survival was reduced following contact with these cloths. A positive correlation between the length of time the cloths were left in the cages and mosquito survival [8] was a strong indication that the mosquitoes pick up inoculum from the cloths when landing on these surfaces in a “time-dose” dependent manner. Under laboratory conditions, Paula and colleagues [7] investigated the dose–response of *A. aegypti* to IMI impregnated surfaces. A non-lethal concentration of 0.1 ppm was established which was then combined with conidia of *M. anisopliae*, resulting in significant decreases in mosquito survival rates when compared to the use of the fungus alone. The aim of that study was to show the advantages of combining both biological and chemical control methods, resulting in significant reductions in vector survival rates without the need to apply high concentrations of chemical insecticides. However, the concentrations of both agents required for effective control under field conditions had not been investigated. Therefore the current study was carried out in rooms simulating intra-domicile conditions.

The one-way ANOVA showed that there were differences among the groups [F _(5, 17)_ = 219.81; p < 0.01] and the Duncan’s test showed that the ESALQ 818 + 10 ppm group had the lowest survival (38%; see Table 1) when compared to all other groups (p < 0.05). Table 1 showed that the ESALQ 818 and ESALQ 818 + 0.1 ppm groups had lower survival rates (44 and 43% respectively) than all the control groups, i.e., control (Tween only), 0.1 ppm only and 10 ppm only groups (p < 0.05). There were no differences in survival rates among these “control” groups (see Table 1; p > 0.05).

**Table 1:** **Survival of female *Aedes aegypti *following a 7 day exposure of mosquitoes to black cloths impregnated with IMI or Fungus + IMI in simulated intra-domicile experiments**

**Treatment**	**% Survival (Mean ± SD)**
CONTROL	76 ± 1.41 a
0.1 ppm	74 ± 1.11 a
10 ppm	73 ± 0.70 a
ESALQ 818	44 ± 1.52 b
ESALQ 818 + 0.1 ppm	43.3 ± 0.57b
ESALQ 818 + 10 ppm	38.3 ± 1.02 c

Note: Results followed by the same letter indicate no significant differences between mean survival when using Duncan’s post-hoc test (5% probability).

Fungus + IMI (0.1 ppm) which was effective in laboratory tests [7] showed no synergist effect on adult survival when compared to the use of cloths impregnated with fungus alone under simulated intra-domicile conditions. Here it was necessary to increase the concentration of IMI to 10 ppm in order to see statistically different survival rates to those observed when the fungus was used alone. Experiments here confirmed that mosquito survival in the presence of black cloths impregnated with 10 ppm IMI alone was not significantly different to that of the Tween only control group (Table 1). The statistical difference between fungus alone and fungus + IMI (10 ppm) confirms the advantage of using this strategy under field conditions. Although it is important to point out that the use of black cloths impregnated with fungus alone also reduced survival to 44% (Table 1).

**Figure 1:**
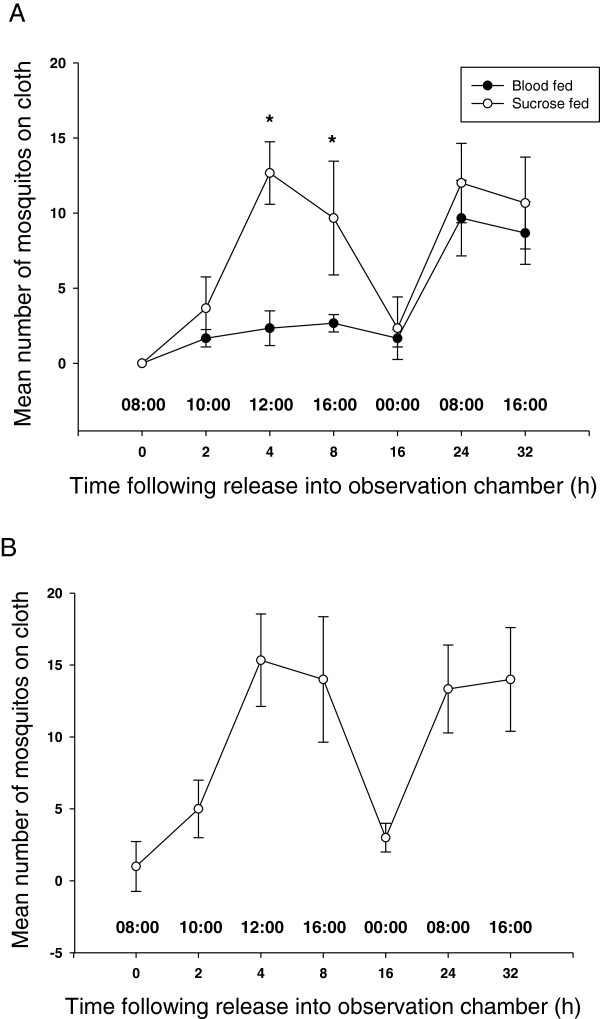
**Behaviour of female mosquitoes in the presence of black cloths. A**. Landing frequency of female *Aedes aegypti* in different nutritional states on black cloths. **B**. Landing frequency of female *Aedes aegypti* on black cloths impregnated with conidia of the entomopathogenic fungus *Metarhizium anisopliae*. Results were mean number (± SD) of mosquitoes observed on black cotton cloths at different times following feeding on blood or sucrose. The X axis also shows chronological time. *Significant differences between number of mosquitoes observed on cloths when comparing sucrose and blood fed insects.

Figure 1A shows the number of sucrose and blood fed mosquitoes resting on the black cloth over a 32 h period post-release into an observation chamber. Lower numbers of blood fed *A. aegypti* were observed on black cloths than sucrose fed insects during the first 24 h following feeding. Sucrose fed insects were observed on the black cloth 4-8 h after being released in the test chamber. This coincided with daylight hours (12-16 pm). Few mosquitoes (4%) were observed on the cloths during the midnight evaluations. The two-way ANOVA showed that there was an interaction between groups and time [F _(6, 42)_ = 5.25; p < 0.01], an effect of groups [F _(1, 42)_ = 31.34; p < 0.01] and an effect of time [F _(6, 42)_ = 25.0; p < 0.01]. To further analyse the interaction between group and time, an independent *t-test* was performed for the groups at each time period and the results showed that there were differences between the sucrose fed and blood fed groups at 4 and 8 h following release into the chamber, with a significantly higher number of sucrose fed mosquitoes resting on the cloth (p < 0.05). For the analysis of the time factor, a one-way ANOVA followed by Duncan’s test showed that for the sucrose fed group at time zero, 2 h and 16 h, the number of mosquitoes on the cloth was lower than for 4, 8, 24 and 32 h (p < 0.05). For the blood fed group the highest numbers of mosquitoes were observed on the cloth (p < 0.05) at 24 and 32 h.

Interestingly, blood-fed insects have been shown to be less susceptible to infection by entomopathogenic fungi than their sucrose fed counterparts [9,10]. This phenomenon could be explained by the reduced contact with fungus impregnated cloths during the 24 h period post-blood feeding, when flight activity could have been lower than that of sucrose fed insects.

Figure 1B shows the number of mosquitoes landing on fungus impregnated black cloths observed over a 32 h period. The temperature range registered during the experiment was 26.9- 29.9°C and relative humidity 72-84%. The results were similar to those seen for cloths without fungus, with the first peak of landing on the cloth (46%) observed 4 h after releasing the insects into the chamber, coinciding with highest natural luminosity levels (12 noon). The numbers of mosquitoes landing on the cloths continued high (42%) at 16:00 h. The next observation was carried out at midnight when few mosquitoes (9%) were observed to be resting on the cloth. A second peak was observed the following day with high percentages of mosquitoes observed on the cloths at 8:00 h and 16:00 h (40% and 42% respectively). The results showed that the number of mosquitoes observed on fungus impregnated cloths was equal to that seen on un-treated cloths (student t-test: t_(40)_ = 1,17; p > 0.05), indicating that the fungus did not repel *A. aegypti*.

Other studies have also shown that fungus impregnated surfaces do not act as repellants to adult mosquitoes [14]. It is interesting to note that very few mosquitoes landed on the cloths during the hours of darkness. Landing on dark surfaces is thought to be a defensive behaviour, as the insects become difficult for predators to detect (RIS: personal observation). We only succeeded in observing these behavioral patterns when subjecting mosquitoes to natural light conditions. The use of standard artificial lighting was not adequate for these types of experiments.

Studies of flight activity of a range of mosquito species suggest that blood meals impose a heavy burden in terms of dispersal ability as non-engorged mosquitoes often fly far greater distances [15]. In *Anopheles atroparvus*, the maximal flight distances were 10–12 km for sugar-fed mosquitoes and 4.5 km when blood-fed [16]. Our results show that even over short distances, blood engorged *A. aegypti* were less likely to land on black cloths, which may have been due to reduced flight activity, although this was not quantified here.

Two studies were carried out recently to test fungus delivery systems for the control of malaria mosquitoes in Africa. In the first study, an intra-domicile method, was tested using fungus treated baffles attached to the eaves of houses or fungus-treated strips of cloth placed around an occupied bed net. These methods resulted in a 39-57% reduction in mosquito survival [17]. In the other study, the use of bait stations was investigated due to the health concerns regarding application of entomopathogenic fungi within human dwellings. The use of odor baited stations containing fungus impregnated cloths was tested against the malaria mosquito *Anopheles arabiensis*, when positioned in rural areas away from human dwellings [18]. The results showed that 95% of mosquitoes that visited the fungus impregnated bait stations died within 14 days.

The first study carried out here using black cloths impregnated with fungus under natural intra-domicile conditions resulted in 44% mosquito survival. Control survival under the same conditions was 76%. With the aim of further reducing survival rates, the insecticide Imidacloprid was mixed with the conidial suspensions. The results obtained using ESALQ 818 + 10 ppm IMI were most promising, with survival rates of 38% recorded.

In Australia, the effect of *B. bassiana* on survival, blood-feeding behaviour and fecundity was tested against *A. aegypti* in large cages (5 × 7 × 4 m), with results showing an 80% reduction in blood-feeding and a reduction in mosquito survival of 59 - 95% [19]. However, the mosquitoes were infected under laboratory conditions and then released into the cages to observe modifications in behaviour and survival. We are currently investigating the persistence/virulence of fungus impregnated cloths under natural conditions with the aim of maintaining efficiency during at least a one month period.

The results presented here show that fungus impregnated black cloths reduce the survival of female *A. aegypti* under simulated intra-domicile field conditions. The use of the fungus combined with the insecticide IMI further reduced mosquito survival. Female *A. aegypti* were observed to land on black cloths during daylight hours. However, shortly following a blood meal, females were less likely to be found on the cloths. No differences in frequency of landing on fungus or non-fungus impregnated cloths was seen, therefore the fungus did not repel insects.

The use of fungus impregnated black cotton cloths has been shown to reduce mosquito survival under simulated intra-domicile conditions, without controlling environmental factors such as temperature and humidity. An understanding of mosquito behaviour in the presence of black cloths will help in planning the use of this point source technique for delivering fungal inoculum in the field.

## Competing interests

The authors declare that they have no competing interests.

## Authors’ contributions

ARP helped to carry out the experiments, participated in the design of the study and performed the statistical analysis. ATC and CRP helped carry out experiments and ATC maintained the insect colonies. CPS participated in the design of experiments and writing of the manuscript. RIS conceived the study, participated in its design, supervised the experiments and wrote the manuscript. All authors read and approved the final manuscript.

## Supplementary Material

Additional file 1 Photographs of room used to test efficiency of fungus impregnated cloths and observation chamber used to study mosquito behaviour in the presence of black cloths. Click here for file
