# Supplementary material for: Testing fungus impregnated cloths for the control of adult Aedes aegypti under natural conditions
Source: Parasit Vectors. 2013 Sep 8;6:256. doi: 10.1186/1756-3305-6-256 (PMC3848359; doi:10.1186/1756-3305-6-256)
Supplement: Additional file 1 — Photographs of room used to test efficiency of fungus impregnated cloths and observation chamber used to study mosquito behaviour in the presence of black cloths. [file 1756-3305-6-256-S1.pdf]

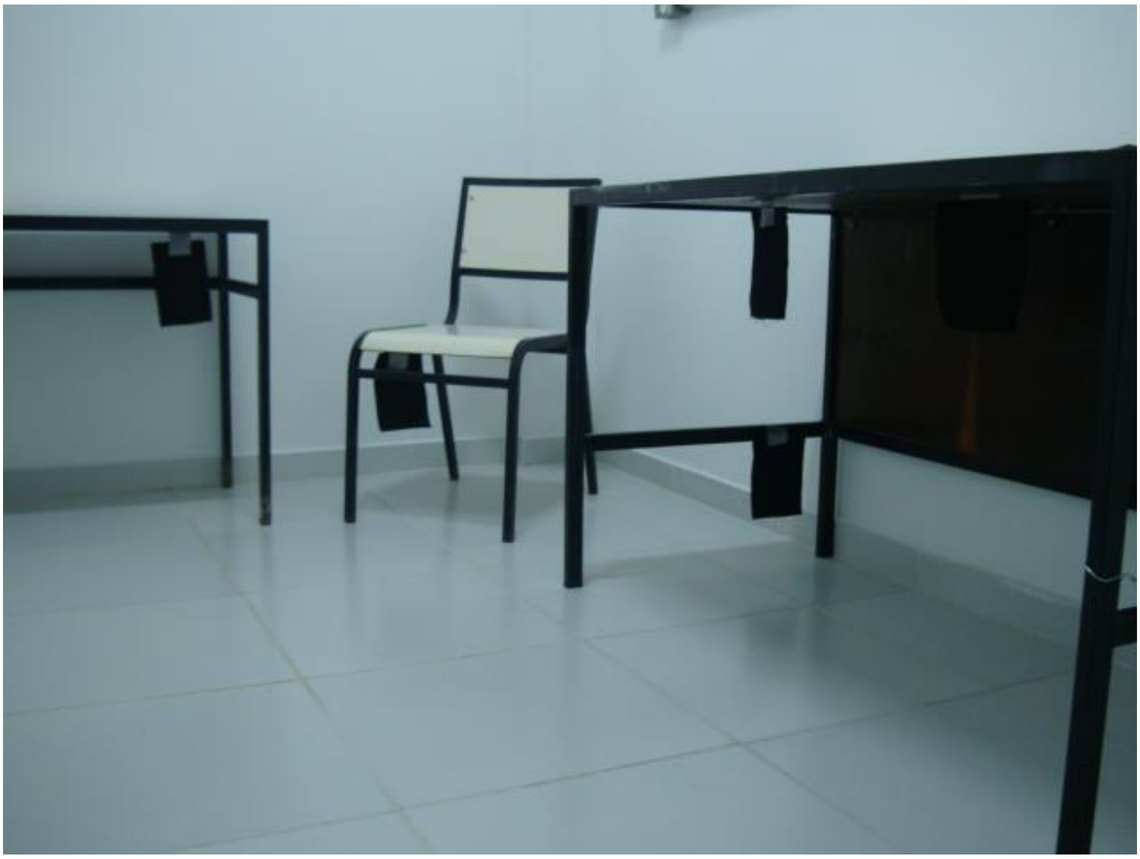

Photo 1: Room used for testing fungus impregnated black cloths.

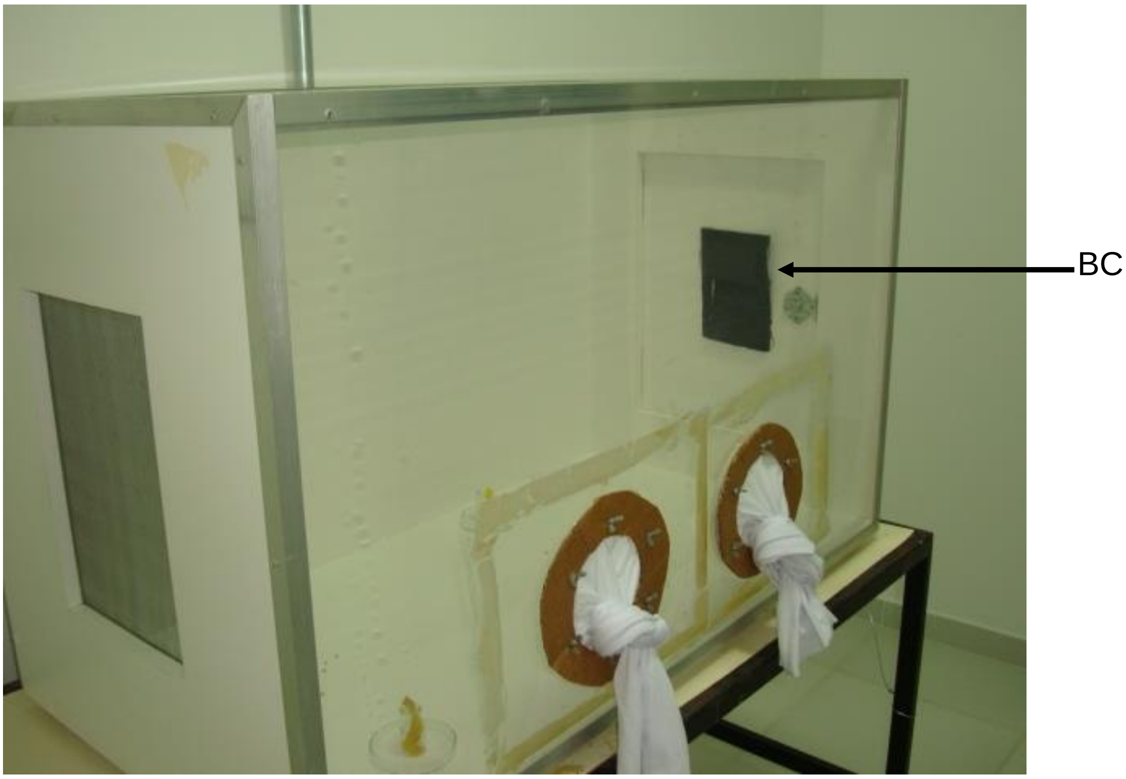

Photo 2: Observation chamber used to monitor mosquito behavior in the presence of black cloths (BC).
